# The AP2/ERF transcription factor SlERF52 functions in flower pedicel abscission in tomato

**DOI:** 10.1093/jxb/eru154

**Published:** 2014-04-17

**Authors:** Toshitsugu Nakano, Masaki Fujisawa, Yoko Shima, Yasuhiro Ito

**Affiliations:** National Food Research Institute, 2-1-12 Kannondai, Tsukuba, Ibaraki 305–8642, Japan

**Keywords:** Abscission, abscission zone, cell-wall hydrolytic enzyme, ERF, functional switching, meristem, tomato, transcription activator, transcription factor.

## Abstract

In plants, abscission removes senescent, injured, infected, or dispensable organs. Induced by auxin depletion and an ethylene burst, abscission requires pronounced changes in gene expression, including genes for cell separation enzymes and regulators of signal transduction and transcription. However, the understanding of the molecular basis of this regulation remains incomplete. To examine gene regulation in abscission, this study examined an ERF family transcription factor, tomato (*Solanum lycopersicum*) *ETHYLENE-RESPONSIVE FACTOR 52* (*SlERF52*). *SlERF52* is specifically expressed in pedicel abscission zones (AZs) and *SlERF52* expression is suppressed in plants with impaired function of *MACROCALYX* and *JOINTLESS*, which regulate pedicel AZ development. RNA interference was used to knock down *SlERF52* expression to show that *SlERF52* functions in flower pedicel abscission. When treated with an abscission-inducing stimulus, the *SlERF52-*suppressed plants showed a significant delay in flower abscission compared with wild type. They also showed reduced upregulation of the genes for the abscission-associated enzymes cellulase and polygalacturonase. *SlERF52* suppression also affected gene expression before the abscission stimulus, inhibiting the expression of pedicel AZ-specific transcription factor genes, such as the tomato *WUSCHEL* homologue, *GOBLET*, and *Lateral suppressor*, which may regulate meristematic activities in pedicel AZs. These results suggest that *SlERF52* plays a pivotal role in transcriptional regulation in pedicel AZs at both pre-abscission and abscission stages.

## Introduction

In plants, organ abscission specifically detaches senescent, injured, infected, or dispensable leaves or flower organs to maintain the healthy growth of the main body. Abscission also detaches mature seeds or fruits to disperse the plant’s progeny. To abscise an organ, plants generally develop a specialized tissue, the abscission zone (AZ), at a predetermined site on the organ to be abscised. Under normal conditions, the AZ firmly attaches the organ to the plant body; after initiation of abscission, the AZ tissues weaken, allowing the organ to detach. Plant hormones act in opposition to regulate organ separation: ethylene promotes abscission and auxin inhibits abscission, in an ethylene-antagonistic manner (Taylor and Whitelaw, 2001; Meir *et al.*, 2010). Abscission involves the activation of cell-wall-degradation machinery in the AZ, including cell-wall hydrolytic enzymes such as endo-β-1,4-glucanase (also referred as cellulase (Cel)), polygalacturonase (PG), expansin, and xyloglucan endotransglucosylase/hydrolase (Roberts *et al.*, 2002; Nakano and Ito, 2013). These enzymes degrade the primary cell wall or middle lamella pectin of AZ tissues so that abscising organs detach easily from the parent plant. Marked changes in transcription activate cell-wall degradation and other abscission processes (Meir *et al.*, 2010; Wang *et al.*, 2013); therefore, unveiling the mechanisms of transcriptional regulation will enable a more clear understanding of the onset of abscission. In *Arabidopsis thaliana*, various transcription factors (TFs) positively or negatively regulate abscission of floral organs, including stamens, petals, and sepals. These TFs include members of the KNOTTED-LIKE HOMEOBOX (KNOX) family, the DNA BINDING WITH ONE FINGER (DOF) family, the MADS-box family, the ETHYLENE-RESPONSIVE FACTOR (ERF) family, the AUXIN RESPONSE FACTOR (ARF) family, and the ZINC FINGER family (Fernandez *et al.*, 2000; Ellis *et al.*, 2005; Cai and Lashbrook, 2008; Wei *et al.*, 2010; Chen *et al.*, 2011; Shi *et al*., 2011*a*,*b*). However, the relationships among these TFs and the resulting transcriptional cascades remain incompletely understood.

Tomato (*Solanum lycopersicum*) plants develop AZs at the midpoint of the flower pedicels. The AZs have a knuckle-like structure with a groove on the surface. If pollination fails, the flower will senesce and eventually abscise from the plant at the AZ. During flower pedicel abscission, expression of *PG* and *Cel* greatly increases (Meir *et al.*, 2010; Nakano *et al.*, 2013; Wang *et al.*, 2013). Programmed cell death also occurs during flower pedicel abscission (Bar-Dror *et al.*, 2011). In tomato, several mutations can inhibit development of pedicel AZs, causing a ‘jointless’ phenotype. For example, *jointless* (*j*) is a mutation of a MADS-box TF gene and *lateral suppressor* (*ls*) is a mutation of a GRAS family TF gene (Schumacher *et al.*, 1999; Mao *et al.*, 2000). The locus for another ‘jointless’ mutation, *j-2*, has not yet been identified, but a sequencing analysis has identified a candidate gene encoding C-terminal domain (CTD) phosphatase-like 1 (*ToCPL1*) (Yang *et al.*, 2005). In addition, the current study group has showed that the MADS-box TF MACROCALYX (MC) regulates pedicel AZ development and that a heterodimer of MC and J functions as a unit for this regulation (Nakano *et al.*, 2012). Recent work identified another tomato MADS-box TF gene, *SlMBP21*, as a regulator of pedicel AZ development and showed that the encoded protein also interacts with MC and J (Liu *et al.*, 2014). To identify more genes involved in pedicel abscission, Nakano *et al.* (2012, 2013) identified genes that are regulated by both *MC* and *J* and are expressed specifically in pedicel AZs. Interestingly, the results of this screen suggested that the tomato *WUSCHEL* homologue (*LeWUS*), *GOBLET* (*GOB*), *Ls*, and *BLIND* (*Bl*), which regulate meristem activity, also regulate pedicel AZ activity. However, their detailed roles in AZs remain unknown. The screen also identified several other TF genes: *OVATE*, *SlERF52*, and a zinc finger-homeodomain (ZF-HD) family protein.

Based on the previous study, the current work focused on an ERF family TF gene, *SlERF52.* The ERF family TFs constitute one of the largest TF families in the plant kingdom (Riechmann *et al.*, 2000); for example, the tomato genome includes at least 85 genes for ERF family proteins, most of which remain uncharacterized (Sharma *et al.*, 2010). The ERF family members contain a single DNA-binding domain, the APETALA2 (AP2)/ERF domain (Ohme-Takagi and Shinshi, 1995), and, as monomers, recognize the GCC-box or CRT/DRE (for C-repeat/dehydration responsive element) *cis*-acting DNA elements (Allen *et al.*, 1998; Hao *et al.*, 1998; Yang *et al.*, 2009). The AP2/ERF domain was identified in proteins binding to ethylene-responsive gene promoters (Ohme-Takagi and Shinshi, 1995), but subsequent studies revealed that the ERF family TFs function in diverse aspects of plant growth, development, and physiology, such as meristem activity, floral organ abscission, lipid metabolism, alkaloid biosynthesis, and responses to environmental stress (extreme temperature, water deficit, salinity, low oxygen, and pathogen infection) (Stockinger *et al.*, 1997; Liu *et al.*, 1998; Solano *et al.*, 1998; van der Fits and Memelink, 2000; Banno *et al.*, 2001; Berrocal-Lobo *et al.*, 2002; Gu *et al.*, 2002; Kirch *et al.*, 2003; Komatsu *et al.*, 2003; Broun *et al.*, 2004; Xu *et al.*, 2006; Shoji *et al.*, 2010; Iwase *et al.*, 2011). The current study used gene suppression to investigate the function of *SlERF52*. The results demonstrate that *SlERF52* is required for activation of cell-wall-degrading enzymes during abscission as well as pedicel-specific gene expression at the pre-abscission stage.

## Materials and methods

### Plant materials

The tomato cultivar Ailsa Craig was used to make transgenic plants. The *jointles*s mutant (TK3043) and the *MC*-suppressed transgenic plants were described previously (Nakano *et al.*, 2012). Plants were grown in a controlled growth room under a 16/8 light/dark cycle at 25 °C.

### Plasmid construction

Oligonucleotide primers used for gene amplification are listed in Supplementary Table S1 (available at *JXB* online). To obtain the *SlERF52* gene fragments, cDNAs were synthesized from flower pedicel total RNA and used as templates for PCR amplification. A plasmid for RNA interference (RNAi) targeting *SlERF52* was constructed as follows. A 315-bp fragment of *SlERF52* was amplified with a pair of gene-specific primers, AK327476-F2 and AK327476-R2, and then cloned into the pENTR/D-TOPO Gateway entry vector (Invitrogen). The cloned fragment was transferred into a binary vector for RNAi, pBI-sense, anti sense-GW (Inplanta Innovations, Japan) using Gateway LR Clonase Enzyme Mix (Invitrogen). The resultant plasmid was designated pBI-GW-SlERF52-RNAi.

Plasmids for the transactivation assay were constructed as follows. The full-length open reading frame of *SlERF52* was amplified with the primer pair NcoI-SlERF52-F1 and BamHI-SlERF52-R1 and inserted into the *Nco*I and *Bam*HI sites of pGBKT7 (Clontech), which carries an auxotrophic marker gene (*TRP1*). The resulting plasmid was designated pGBK-SlERF52. Sequencing analysis revealed that *SlERF52* from Ailsa Craig possesses five single-nucleotide polymorphisms in comparison with the genome sequence of the cultivar Heinz 1706 (accession no. AB889741). A series of partial *SIERF52* fragments were amplified using NcoI-SlERF52-F1 and BamHI-SlERF52-R2 for amino acids 1–74, NcoI-SlERF52-F1 and BamHI-SlERF52-R3 for amino acids 1–98, NcoI-SlERF52-F1 and BamHI-SlERF52-R4 for amino acids 1–133, and NdeI-SlERF52-C3 and BamHI-SlERF52-R1 for amino acids 133–162. Each amplified DNA fragment was inserted into pGBKT7, resulting in pGBKT7-SlERF52_1–74_, pGBKT7-SlERF52_1–98_, pGBKT7-SlERF52_1–133_, and pGBKT7-SlERF52_133–162_, respectively.

### Plant transformation

The plant transformation vector pBI-GW-SlERF52-RNAi was introduced into *Agrobacterium tumefaciens* EHA105 by the freeze–thaw method (Cindy and Jeff, 1994). Cotyledons of tomato seedlings were used for transformation by *Agrobacterium* infection according to the previously described method (Sun *et al.*, 2006).

### Transactivation assay

Transactivation assays in yeast cells were conducted according to the previously described method (Cho *et al.*, 1999). The yeast strain AH109 (Clontech), which carries two auxotrophic marker genes (*ADE2* for adenine biosynthesis and *HIS3* for histidine biosynthesis) under the GAL4 *cis*-regulatory element, was used for the experiment. Yeast transformation was performed using the Frozen EZ Yeast Transformation II kit (Zymo Research, Irvine, CA, USA), and transformants were selected on SD media lacking tryptophan (SD/–Trp, Clontech). Assays for transactivation activity were performed on SD media lacking tryptophan, adenine, and histidine (SD/–Trp/–Ade/–His). In the experiment, a target protein was expressed as a fusion protein with the GAL4 DNA-binding domain (GAL4DBD), and if the protein had the potential to activate transcription, the auxotrophic marker genes (*ADE2* and *HIS3*) were expressed and the yeast cell was able to grow on the adenine- and histidine-deficient selection medium.

### Sequence analysis

Multiple sequence alignment was performed with ClustalW version 1.83 and the phylogenetic tree was constructed by the neighbour-joining method. GENETYX version 10 (GENETYX, Japan) was used for the analysis. Supplementary Table S2 shows the accession numbers for the sequences used in the analysis.

### Reverse-transcription PCR and quantitative reverse-transcription PCR

Total RNAs were extracted using the RNeasy plus mini kit (Qiagen) in combination with the QIA shredder spin column (Qiagen). First-strand cDNA was synthesized using PrimeScript II 1st strand cDNA Synthesis Kit (Takara Bio, Japan). PCR amplifications were performed using the ExTaq polymerase (Takara Bio). qRT-PCR was carried out with a 7300 Real-Time PCR System (Applied Biosystems) using THUNDERBIRD SYBR qPCR MIX (Toyobo, Japan). Data were normalized to the expression of the *SAND* gene (SGN-U316474) as an internal control (Exposito-Rodriguez *et al.*, 2008). Relative quantification of expression of each gene was performed using the 2^–ΔΔCT^ method (Livak and Schmittgen, 2001).

### Flower pedicel abscission assay

Flower pedicels were harvested at anthesis. The flower was removed from the pedicel using a sharp blade, the pedicel end was inserted into a 1.0% agar plate, and the plate was placed in a glass chamber to maintain high humidity. An abscission event was defined by pedicel detachment that occurred naturally or in a response to vibration applied to the distal portion of the explant.

## Results

### SlERF52 is a member of the ERF transcription factor family

**Fig. 1.**
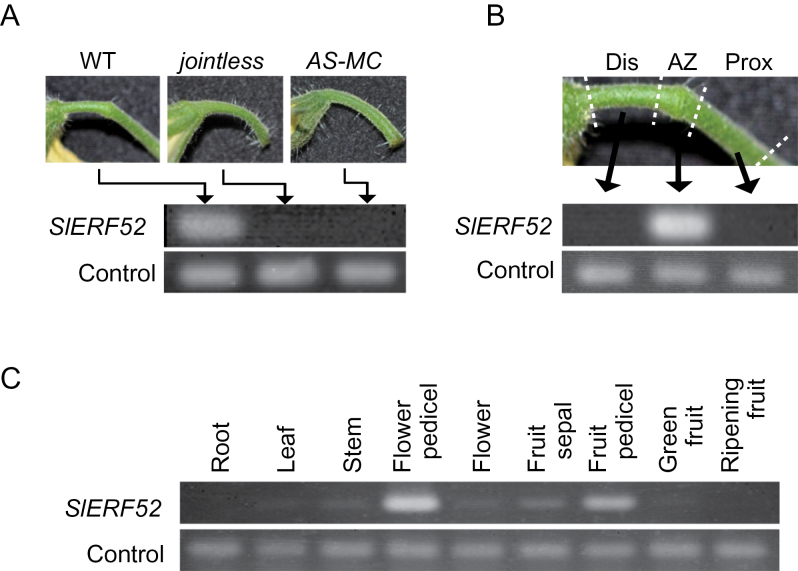
Expression specificity of *SlERF52*. (A) Expression analysis of *SlERF52* in a *jointless* mutant, a *MC*-suppressed transgenic plant (*AS-MC*), and the wild type (WT). (B) Expression specificity of *SlERF52* within flower pedicel parts, the distal (Dis), proximal (Prox), and abscission zone (AZ) regions in WT anthesis flowers. (C) Expression analysis of *SlERF52* among various organs. Expression analysis was performed by reverse-transcription PCR using *SAND* (A and B) and *SlActin-51* (C) as the internal control.

As described previously, *SlERF52* expression is strictly limited to the AZ region in the pedicel and *SlERF52* expression is suppressed in plants that lack an AZ, namely *MC-*knockdown plants and *j* mutants (Nakano *et al.*, 2012, 2013; Fig. 1A and B). No or very low expression of *SlERF52* was detected in other organs, including roots, leaves, stems, flowers, sepals, and fruits (Fig. 1C). These results suggest that *SlERF52* plays a specific role in pedicel abscission.

**Fig. 2.**
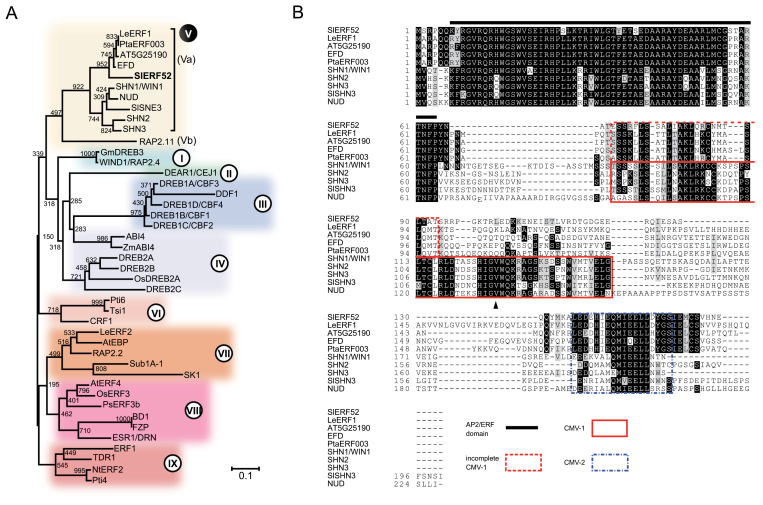
Phylogenetic analysis and sequence alignment of SlERF52. (A) Phylogenetic relationships of SlERF52 with ERF proteins; the phylogenetic tree was constructed using amino acid sequences of the AP2/ERF domains in each ERF protein (Supplementary Table S2). (B) Multiple sequence alignment of group Va ERF proteins. Following the highly conserved AP2/ERF domain, there are two conserved motifs, CMV-1 and CMV-2 (Nakano *et al.*, 2006). Based on the CMV-1 structure, the group Va ERFs are further classified into two subgroups: the normal CMV-1 subgroup including *Arabidopsis* SHNs, SlSHN3, and NUD and the incomplete CMV-1 subgroup including SlERF52, LeERF1, PtaERF003, AT5G25190, and EFD. The CMV-2 motif is conserved in both subgroups. Arrowhead indicates the amino acid substituted in the *nud* mutant (*nud1.b*; Taketa *et al.*, 2008).

Phylogenetic analysis of the AP2/ERF domain revealed that SlERF52 belongs to group Va of ERFs (Fig. 2A). This group includes: *Arabidopsis* WAX INDUCER 1 (WIN1)/SHINE1 (SHN1), SHN2, and SHN3, which regulate cutin biosynthesis and abscission of floral organs (Aharoni *et al.*, 2004; Broun *et al.*, 2004; Shi *et al.*, 2011*b*); the tomato homologue of SHN3 (SlSHN3) (Shi *et al.*, 2013); barley (*Hordeum vulgare*) NUDUM (NUD), which regulates lipid biosynthesis for hull-caryopsis adhesion of grain (Taketa *et al.*, 2008); tomato LeERF1, which regulates ethylene signalling (Li *et al.*, 2007); *Medicago truncatula* ERF REQUIRED FOR NODULE DIFFERENTIATION (EFD) (Vernie *et al.*, 2008); and poplar (*Populus tremula × P. alba*) PtaERF003, which is involved in adventitious and lateral root formation (Trupiano *et al.*, 2013). Group Va ERFs have three conserved domains: the AP2/ERF domain, conserved motif V (CMV)-1, and CMV-2 at the C-terminus (Fig. 2B). Group Va includes two subgroups, a subgroup with the normal CMV-1 domain (including WIN1/SHN1 and its orthologues), and another subgroup with an incomplete CMV-1 domain (including LeERF1, the AT5G15190-encoding protein, EFD, PtaERF003, and SlERF52; Fig. 2B).

### SlERF52 acts as a positive regulator of flower pedicel abscission

**Fig. 3.**
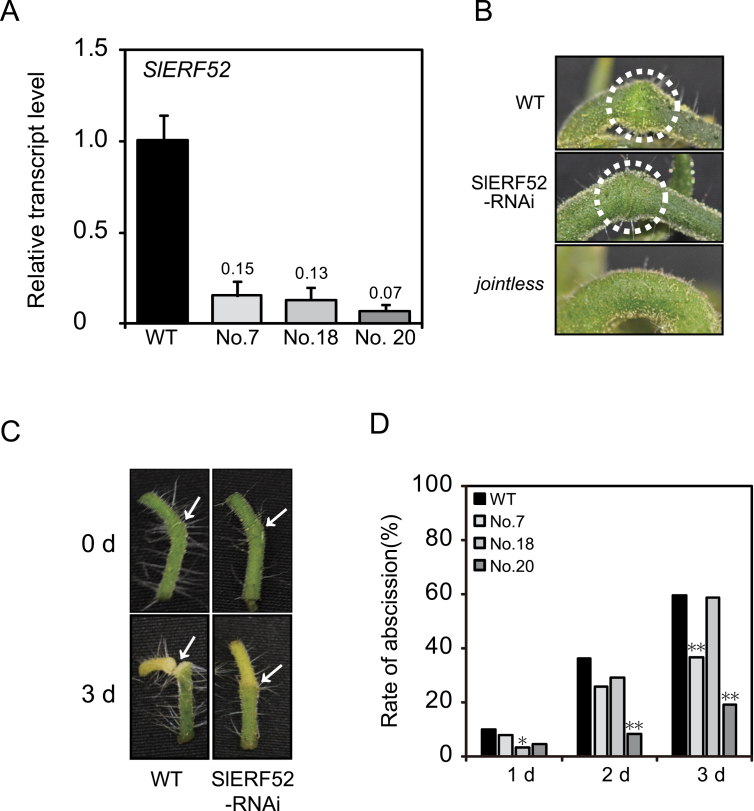
Suppression of *SlERF52* partially inhibited flower pedicel abscission. (A) Transcript levels of *SlERF52* in three *SlERF52*-suppressed transgenic plants (plants 7, 18, and 20). Transcript level was examined in anthesis flower pedicels by quantitative reverse-transcription PCR. The levels are shown as fold-change values relative to that of WT; error bars indicate standard deviation of biological triplicates. (B) Suppression of *SlERF52* did not affect pedicel abscission zone development. (C) Pedicel abscission was inhibited in the *SlERF52*-suppressed transgenic plants; arrows indicate the abscission zone. (D) Rate of flower pedicel abscission in *SlERF52*-suppressed transgenic plants. Pedicel abscission was induced by removing anthesis flowers. Asterisks indicate significant differences by chi-square test (*P<0.05, **P<0.01, respectively; *n*=206 for WT, *n*=112 for plant 7, *n*=119 for plant 18, and *n*=110 for plant 20).

To analyse the biological role of *SlERF52*, RNAi was used to knock down *SlERF52* expression. To that end, transgenic plants with an RNAi vector targeting *SlERF52* were generated, 15 independent transgenic plants were obtained, and the three plants with the lowest expression levels of *SlERF52* (plants 7, 18, and 20) were selected for further analysis (Fig. 3A and Supplementary Fig. S1). The three *SlERF52*-suppressed plants appeared similar to wild-type plants and developed pedicel AZs normally (Fig. 3B), indicating that *SlERF52* does not regulate differentiation of pedicel AZs. To examine the pedicel abscission behaviour of the transgenic plants, flower pedicel abscission was induced by removing the flower from the pedicel, which stimulates ethylene production and restricts auxin supply from the flower (Meir *et al.*, 2010) and observing the frequency of abscission in the flower-removed pedicels for 3 d (Fig. 3C). The abscission frequency of pedicels from plants 7 and 20 at 3 d after flower removal was significantly lower than that of wild type, indicating that the pedicels of the two suppression lines showed decreased abscission potential compared to the wild type (Fig. 3D). The pedicels from plant 18 exhibited significant reduction of abscission frequency at 1 d after flower removal, although the abscission eventually occurred at the same level as the wild type at 3 d after flower removal (Fig. 3D). These observations indicate that the suppression of *SlERF52* impaired activation of pedicel abscission.

### Suppression of *SlERF52* inhibits induction of genes for cell-wall hydrolytic enzymes

**Fig. 4.**
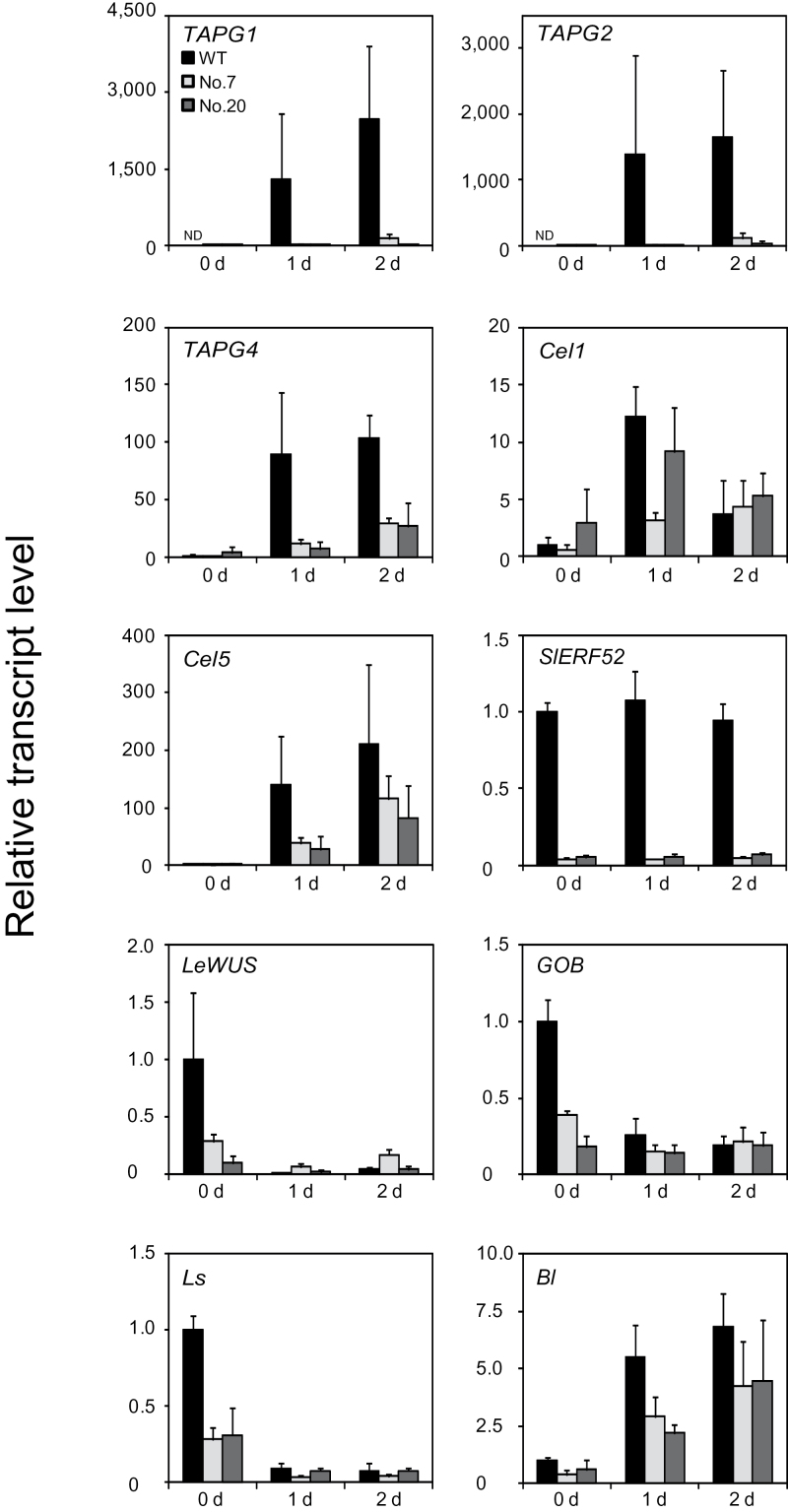
Expression analysis in *SlERF52*-RNAi plants during abscission. Pedicel abscission was induced by anthesis flower removal and gene expression was investigated for 2 d by quantitative reverse-transcription PCR. For single RNA sample preparation, 3–24 pedicel abscission zones, which include both attached and abscised pedicels, were harvested in bulk and used for the analysis. Levels of transcripts are shown as fold-change values relative to the 0 d sample of WT (for *Cel1*, *Cel5*, *TAPG4, SlERF52*, *Bl*, *GOB*, *LeWUS*, and *Ls*). Because *TAPG1* and *TAPG2* transcript levels for the 0 d sample of WT were below detection limit (shown as ND), the level of the two genes are shown relative to the sample of *SlERF52*-suppressed plant 20 at 1 d. Data are mean±SD of biological triplicates.

Expression of genes encoding cell-wall hydrolytic enzymes, including PG and Cel, is induced in response to the abscission stimulus (Roberts *et al.*, 2002). Because suppression of *SlERF52* decreased the rate of pedicel abscission, the current work investigated whether it also affected the transcript levels of genes encoding PG (*TAPG1*, *TAPG2*, and *TAPG4*) and Cel (*Cel1* and *Cel5*) during flower pedicel abscission. In accord with previous reports (Meir *et al.*, 2010; Nakano *et al.*, 2013; Wang *et al.*, 2013), in wild-type plants, removal of the flower induced the expression of *TAPG1*, *TAPG2, TAPG4*, *Cel1*, and *Cel5* in AZs, but *SlERF52* was expressed at constant levels before and after the onset of abscission (Fig. 4). In *SlERF52*-suppressed plants 7 and 20, *TAPG1*, *TAPG2*, *TAPG4*, and *Cel5* were induced to significantly lower levels than in the wild type (Fig. 4), and the levels of these four genes corresponded to the abscission rates in the suppressed transformants (Fig. 3). The suppression was more severe for *PG* genes than for *Cel5*. Meanwhile, the levels of *Cel1* expression did not correspond to the abscission rate.

### Suppression of *SlERF52* reduces expression of transcription factor genes *LeWUS*, *GOB*, and *Ls* in pedicel AZs

Previously, this study group reported that *LeWUS*, *GOB*, *Ls*, and *Bl*, four TF genes associated with shoot apical meristem or axillary meristem function, might also be involved in the regulation of pedicel AZ activity (Nakano *et al.*, 2012, 2013). To investigate whether *SlERF52* affects the expression of these four TF genes, their transcript levels in the *SlERF52*-suppressed plants were analysed. As observed previously, in wild-type plants, the expression of *LeWUS*, *GOB*, and *Ls* decreased markedly in response to flower removal, an abscission stimulus. In the *SlERF52*-suppressed plants, however, the transcript levels of these three genes were much lower than the wild type before flower removal (0 d) and their levels remained low after flower removal (1 d and 2 d) (Fig. 4). By contrast, the expression of *Bl* increased during abscission similarly in the *SlERF52*-suppressed plants and the wild type (Fig. 4). The transcript level of *Bl* in the suppressed plants was slightly lower than that in wild type throughout the examined period but the difference was not significant, except in the d-1 samples. The expression pattern of these four TF genes was not correlated with the expression of *SlERF52* in shoot apices or leaf axillae of wild type plants and also was not affected by suppression of *SlERF52* (Supplementary Fig. S2), which is consistent with the normal vegetative growth of the suppressed plants. The results suggest that the *SlERF52*-mediated regulation of *LeWUS*, *GOB*, and *Ls* is specific to pedicel AZs.

### SlERF52 functions as a transcriptional activator

**Fig. 5.**
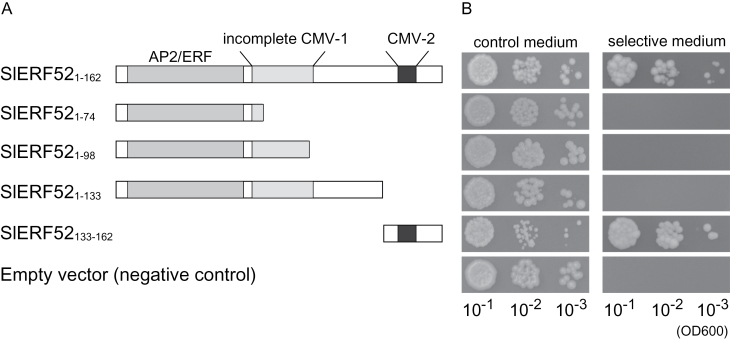
SlERF52 functions as a transcriptional activator. (A) Schematic of full-length and truncated SlERF52 proteins examined in this assay; fused products of these proteins with GAL4DBD were expressed in yeast cells. (B) Results of transactivation assays; yeast cells expressing the fusion proteins were inoculated on SD/–Trp (control medium) and SD/–Trp–His–Ade (selection medium). Yeast cells expressing GAL4DBD were used as a negative control.

ERF proteins can activate or repress transcription of target genes (Fujimoto *et al.*, 2000; Ohta *et al.*, 2001). This study investigated the transcriptional activation potential of SlERF52 using a yeast system, with the GAL4 DNA-binding domain (DBD) fused to SlERF52 and marker genes expressed under the control of the GAL4 target-binding site. The results showed that the construct with the full-length SlERF52 coding region (GAL4DBD-SlERF52_1–162_) induced expression of the marker genes (Fig. 5), indicating that SlERF52 can activate transcription. To identify which region of SlERF52 is necessary for the activity, three truncated SlERF52 proteins (SlERF52_1–74_, SlERF52_1–98_, and SlERF52_1–133_) were assayed, but no activity was detected in any of the C-terminal truncated proteins (Fig. 5). By contrast, this work did detect activity in a construct with the C-terminal 30 amino acids (GAL4DBD-SlERF52_133–162_) (Fig. 5). These results indicated that the transcriptional activation activity of SlERF52 requires the C-terminal 30-amino-acid region that contains the CMV-2 motif.

## Discussion

### SlERF52 functions as a positive regulator of flower pedicel abscission

These data showed that suppression of *SlERF52* reduced the rate of pedicel abscission and repressed induction of the genes for cell-wall hydrolytic enzymes PG and Cel (*Cel5*, *TAPG1*, *TAPG2*, and *TAPG4*). Abscission of flower pedicels and leaf petioles in tomato requires the activity of these enzymes (Lashbrook *et al.*, 1998; Jiang *et al.*, 2008). Therefore, these results suggest that *SlERF52* induces pedicel abscission through upregulation of these enzyme genes. In contrast to the low induction of *Cel5*, *TAPG1*, *TAPG2*, and *TAGP4* in the suppressed plants, the expression of *Cel1* was not correlated with suppression of *SlERF52* or the abscission rate. These results also indicate that the transcript level of *Cel1* in the wild type peaked at 1 d after flower removal and then declined, but the transcript levels of *Cel5*, *TAPG1*, *TAPG2*, and *TAPG4* continuously increased (Fig. 4). In addition, *Cel1* is expressed in a pedicel region distinct from the region where *TAPG1* and *TAPG4* are expressed (Bar-Dror *et al.*, 2011). These results imply that the transcriptional regulation of *Cel1* is independent of the regulation mediated by *SlERF52*. Therefore, these results indicate that *SlERF52* acts as a key positive regulator of flower pedicel abscission, but abscission also involves a *SlERF52*-independent pathway.

Interestingly, SlERF52 is necessary, but not sufficient, for the upregulation of *PG* and *Cel* genes; before the onset of abscission, *SlERF52* is also expressed at a similar level to that observed after flower removal, but this expression does not induce *PG* and *Cel* gene expression (Fig. 4). Post-transcriptional regulation may explain the transcription-independent activity of *SlERF52* (as will be discussed).

SlERF52, a positive regulator of abscission, has an opposite role to that of the *Arabidopsis* homologues, SHNs, which act as negative regulators of abscission of floral organs such as sepals, stamens, and petals (Shi *et al.*, 2011*b*). Simultaneous suppression of all *SHN* genes induces earlier abscission of floral organs, possibly due to decreased cutin deposition and altered cell-wall composition of structural proteins and pectin (Shi *et al.*, 2011*b*). SlERF52 and SHNs belong to the group Va ERF family, but belong to different subgroups based on their CMV-1 motif structures: SlERF52 belongs to the subgroup with an incomplete CMV-1, and the SHNs belong to the subgroup with normal CMV-1 structure (Fig. 2B). The biological function of CMV-1 has not been identified, but the structural difference in CMV-1 between SlERF52 and SHNs may be a possible cause of their functional diversity. Also, the latter half of the CMV-1 motif, which is lost in the incomplete-type Va ERFs, contains an important active site, as demonstrated in a study of mutants of *NUD*, a barley orthologue of *WIN1/SHN1* (Fig. 2B; Taketa *et al.*, 2008). Elucidation of the function of the CMV-1 motif will provide insights into the functional diversity between the subgroups within the Va ERFs, including *SlERF52* and *SHNs*.

Several group Va ERF proteins, including WIN1/SHN1, SHN2, SHN3, and EFD, act as transcriptional activators (Vernie *et al.*, 2008; Shi *et al.*, 2011*b*). However, the domain for transcriptional activation was not identified. The current study demonstrated that the C-terminus of SlERF52, which contains the CMV-2 motif, acts as an activation domain. As shown in Fig. 2B, the CMV-2 motif is highly conserved in WIN1/SHN1, SHN2, SHN3, and EFD, suggesting that the conserved motif functions as a transcription activation domain in these proteins.

### SlERF52 is involved in the expression of TF genes for shoot apical meristem and axillary meristem function in flower pedicel AZs

*LeWUS*, *GOB*, *Ls*, and *Bl*, key TF genes for meristem-associated functions, are expressed specifically in flower pedicel AZs, suggesting that these four TFs may have an additional function in control of organ abscission through regulation of meristem-like activity in the cells within the AZ (Nakano *et al.*, 2012, 2013). The current study found that *LeWUS*, *GOB*, and *Ls* were expressed at significantly lower levels in the *SlERF52*-suppressed plants, implying that *SlERF52* may be involved in the regulation of these TF genes. Expression of *SlERF52, LeWUS, GOB*, and *Ls* is reduced in pedicels of *MC*-suppressed plants, *SlMBP21*-suppressed plants, and *j* mutants (Nakano *et al.*, 2012; Liu *et al.*, 2014; Fig. 1A), indicating that *SlERF52* may mediate the effect of *MC*, *J*, and *SlMBP21* on these meristem-associated regulators. Two *SlERF52* homologues that belong to the incomplete CMV-1 type subgroup regulate plant development through modulation of meristem activity: medicago *EFD* controls formation of root nodule meristems (Vernie *et al.*, 2008) and poplar *PtaERF003* controls formation and growth of adventitious and lateral root meristems (Trupiano *et al.*, 2013). Therefore, the control of meristem-associated regulation may be a conserved biological function for the group Va ERFs with incomplete CMV-1 motifs. *PtaERF003* functions in an auxin-regulated pathway that regulates root meristems (Trupiano *et al.*, 2013). Similar to root meristem regulation, expression of the shoot meristem-associated TF genes in the AZs may be regulated by a signalling pathway that requires auxin supplied from the flower before the onset of abscission, and *SlERF52* may function in the auxin signalling pathway in the AZs.

The expression analyses revealed that SlERF52 activates the expression of *LeWUS*, *GOB*, and *Ls* in the AZ cells, but the expression of these three TF genes was suppressed after stimulation of abscission, even though *SlERF52* expression remained constant (Fig. 4). By contrast, the cell-wall hydrolytic enzyme genes were suppressed before the stimulation of abscission, even though *SlERF52* expression remained constant, a reverse pattern to that of the three TF genes. This partial dependence on *SlERF52* is discussed in the next section.

Of the four TF genes for meristem-associated functions, *Bl* exhibits significant upregulation after flower removal, an expression pattern distinct from *LeWUS, GOB*, and *Ls* (Fig. 4). Thus, Nakano *et al.* (2013) hypothesized that an independent pathway controls *Bl* expression, although *MC* and *J* are involved in the expression of all four TF genes. In the current study, the suppression of *SlERF52* did not significantly affect *Bl* expression, indicating that a *SlERF52*-independent pathway regulates *Bl*. Also, the intense induction of *Bl* after flower removal suggests that *Bl* may be involved in pedicel abscission (Nakano *et al.*, 2013). The induction of *Bl* in the *SlERF52-*suppressed plants may help explain the partial progression of abscission in the suppressed lines.

### Functional switching of SlERF52 before and after the onset of abscission

*SlERF52* functions in the regulation of pedicel abscission and regulates transcription of distinct sets of genes before and after the onset of abscission. In the pre-abscission stage, the expression of *LeWUS*, *GOB*, and *Ls* requires *SlERF52*, either directly or indirectly. In response to an abscission-inducing stimulus, the expression of *Cel5*, *TAPG1, TAPG2*, and *TAPG4* was also regulated by *SlERF52*, directly or indirectly. However, after the onset of abscission, the induction of *LeWUS*, *GOB*, and *Ls* ceases. To explain how SlERF52 is involved in the regulation of distinct sets of genes before and after the onset of abscission, it is postulates that coregulators specify the function of SlERF52 in the different states. In this hypothesis, SlERF52 recruits state-specific TFs and each state-specific TF complex activates expression of a distinct set of target genes. Several ERFs are predicted to require cofactors to bind target genes (Chakravarthy *et al.*, 2003; Kannangara *et al.*, 2007; Cheng *et al.*, 2013). As another possibility, repressor proteins or chromatin remodelling at SlERF52-binding sites may restrict the transactivation activity of SlERF52 in a stage-specific manner.

This work used knockdown experiments to examine *SlERF52* function. A recent study using overexpression of *SlMBP21* provided substantial insights on *SlMBP21* gene function, adding to the results of the knockdown assay (Liu *et al.*, 2014). However, unlike the study of *SlMBP21*, overexpression of *SlERF52* may not be effective to clarify SlERF52 function because the activity of SlERF52 in AZs is likely determined by other factors associated with SlERF52, not by the expression level of *SlERF52*.

In conclusion, the results of this study demonstrated that *SlERF52* regulates pedicel AZ-specific transcription at both pre-abscission and abscission stages and that the regulation during the latter stage includes some of the genes required for abscission. The functional switching between before and after the onset of abscission, by a still-unknown mechanism, raises the possibility that SlERF52 serves as a hub TF that regulates the phase transition between the two stages. The identification of the switching mechanism will further improve the understanding of abscission.

## Supplementary material

Supplementary data are available at *JXB* online.

Supplementary Table S1. Sequences of the oligonucleotide primers used in this study.

Supplementary Table S2. Accession numbers of ERFs used for construction of the phylogenetic tree.

Supplementary Fig. S1. Expression analysis of *SlERF52*-RNAi transgenic plants.

Supplementary Fig. S2. Expression of *SlERF52* and meristem-associated TF genes in shoot apex and leaf axilla.

Supplementary Data
