# Supplementary material for: The AP2/ERF transcription factor SlERF52 functions in flower pedicel abscission in tomato
Source: J Exp Bot. 2014 Apr 17;65(12):3111–9. doi: 10.1093/jxb/eru154 (PMC4071829; doi:10.1093/jxb/eru154)
Supplement: Supplementary Data [file supp_65_12_3111__index.html]

The AP2/ERF transcription factor SlERF52 functions in flower pedicel abscission in tomato — The AP2/ERF transcription factor SlERF52 functions in flower pedicel abscission in tomato — Supplementary Data 

# The AP2/ERF transcription factor SlERF52 functions in flower pedicel abscission in tomato

## Supplementary Data

Data files

**Files in this Data Supplement:**

- Supplementary Data - Supplementary Data
