# Supplementary material for: The AP2/ERF transcription factor SlERF52 functions in flower pedicel abscission in tomato
Source: J Exp Bot. 2014 Apr 17;65(12):3111–9. doi: 10.1093/jxb/eru154 (PMC4071829; doi:10.1093/jxb/eru154)
Supplement: Supplementary Data [file supp_eru154_jexbot116145_file001.pdf]

Supplementary data

Manuscript title: The AP2/ERF transcription factor SlERF52 functions in flower pedicel abscission in tomato

Authors: Toshitsugu Nakano, Masaki Fujisawa, Yoko Shima, Yasuhiro Ito

Table S1 Sequences of the oligonucleotide used in this study

Oligo name	Oligo sequence (5'→3')	Description	Gene name	Accession number (Tomato gene ID)
AF426174-F	GGTAATTGGATTGCACTTCCTCA	RT-PCR and qRT-PCR analysis	*BI*	AF426174
AF426174-R	TGCGCCGCTATAATTGACC			(Solyc11g069030)
FJ435163-F2	AACAAATGTGAACCTTGGGAAC	RT-PCR and qRT-PCR analysis	*GOB*	FJ435163
FJ435163-R	CGATCACGTAGACTGAAGAAATACC			(Solyc07g062840)
AJ538329-F2	GGTTATGGAACTTTGGCTATGGAG	RT-PCR and qRT-PCR analysis	*LeWUS*	AJ538329
AJ538329-R	GAAAAGGGTAAGTTGCTGGAGAAG			(Solyc02g083950)
AF098674-F2	CCATCTCGTCTTGGCGTTG	RT-PCR and qRT-PCR analysis	*Ls*	AF098674
AF098674-R	TCACTCCACGCGTTTTCTTC			(Solyc07g066250)
AK327476-F	CCATGTCTCGACCACAACAA	RT-PCR and qRT-PCR analysis	*SlERF52*	AK327476, AB889741
AK327476-R	CATAGGTTCCCAGCCATATCC			(Solyc03g117130)
U13054-F	CACATCCAAGATTAAAGCCTTTGC	qRT-PCR analysis	*Cel1*	U13054
U13054-R	GGCTCTATGGTGAAGTTTTGTTGG			(Solyc08g081620)
AF077339-F	GGGATGACAAGAGACCTGGAA	qRT-PCR analysis	*Cel5*	AF077339
AF077339-R	GCCTGGAATCAATGAGCAGA			(Solyc08g083210)
U23053-F	GCTCGGGTATAAAGATAAGTGATGTG	qRT-PCR analysis	*TAPG1*	U23053
U23053-R	TGTTATGCCGCTACATGGATTAC			(Solyc02g067630)
U70480-F	GGGTTCTGGTATAAAGATAAGCGATA	qRT-PCR analysis	*TAPG2*	U70480
U70480-R	TCAAGTGTTATGCCGCTGCAC			(Solyc02g067640)
U70481-F	GTGCTAGTCTTTGGGCTTGC	qRT-PCR analysis	*TAPG4*	U70481
U70481-R	TCATCGCGTTATGACAACCA			(Solyc12g096750)
SAND-F	TTGCTTGGAGGAACAGACG	RT-PCR and qRT-PCR analysis	*SAND*	SGN-U316474
SAND-R	GCAAACAGAACCCCTGAATC			(Solyc03g115810)
SlActin-51-F	TGTCCCTATTTACGAGGGTTATGC	RT-PCR analysis	*SlActin-51*	Q96483
SlActin-51-R	CAGTTAAATCACGACCAGCAAGAT			(Solyc11g005330)
AK327476-F2	CACC ATGCAACAGAATCTTCAAGG	RNAi vector construction	*SlERF52*	
AK327476-R2	AGCTAATTATTGCATTTATG			
NcoI-SlERF52-F1	TGACCATGGCTCGACCACAACA	Transactivation assay	*SlERF52*	
BamHI-SlERF52-R1	TCGGGATCCTTATTCATTGTGAACAGA			
BamHI-SlERF52-R2	TCGGATCCTTACAGAAACCTTGAAGATTC	Transactivation assay	*SlERF52*$_{1-74}$	
BamHI-SlERF52-R3	TCGGATCCTTATCCAGGTCTTCTTGACG	Transactivation assay	*SlERF52*$_{1-98}$	
BamHI-SlERF52-R4	TCGGATCCTTACATGTATTGTTGTGATGC	Transactivation assay	*SlERF52*$_{1-133}$	
NdeI-SlERF52-C3	CTTGAGGGCATATGAAGGCACTTGAAG	Transactivation assay	*SlERF52*$_{133-162}$	

The restriction enzyme recognition sites added for cloning procedure are indicated by underline.

Table S2 Amino acid sequences of the AP2/ERF domains used for construction of the phylogenetic tree

Protein name	Scientific name	Accession number
LeERF1	*Solanum lycopersicum*	AAL75809
PtaERF003	*Populus trichocarpa*	Potri.018G021900
AT5G25190	*Arabidopsis thaliana*	AAK74017
EFD	*Medicago truncatula*	ABY83189
SlERF52	*S. lycopersicum*	AB889741
WIN1/SHN1	*A. thaliana*	AAR20494
NUD	*Hordeum vulgare*	BAG12386
SlSHN3	*S. lycopersicum*	SGN-U565667
SHN3	*A. thaliana*	AAO63881
SHN2	*A. thaliana*	AY560865
RAP2.11	*A. thaliana*	AAC49777
GmDREB3	*Glycine max*	NP_001236953
WIND1/RAP2.4	*A. thaliana*	AAN12993
DEAR1/CEJ1	*A. thaliana*	AAK73937
CBF3/DREB1A	*A. thaliana*	BAA33791
DDF1	*A. thaliana*	AAT44959
CBF4/DREB1D	*A. thaliana*	AAT44924
CBF1/DREB1B	*A. thaliana*	AAC49662
CBF2/DREB1C	*A. thaliana*	BAA33793
ABI4	*A. thaliana*	AAT44957
ZmABI4	*Zea mays*	AAM95247
DREB2A	*A. thaliana*	BAA33794
DREB2B	*A. thaliana*	BAA33795
OsDREB2A	*A. thaliana*	AF300971
DREB2C	*A. thaliana*	AAM61256
Pti6	*S. lycopersicum*	AAC49741
Tsi1	*Nicotiana tabacum*	AAC14323
CRF1	*A. thaliana*	AAT44916
LeERF2	*S. lycopersicum*	AAO34704
AtEBP	*A. thaliana*	AAN13131
RAP2.2	*A. thaliana*	AAC49768
Sub1A-1	*O. sativa*	AAZ06207
SK1	*O. sativa*	BAH96566
AtERF4	*A. thaliana*	BAA32421
OsERF3	*O. sativa*	BAB16083
PsERF3b	*Prunus salicina*	ACM49844
BD1	*Zea mays*	AAO21119
FZP	*O. sativa*	AAX83538
ESR1/DRN	*A. thaliana*	AAL56226
ERF1	*A. thaliana*	AAD03544
TDR1	*A. thaliana*	CAE45639
NtERF2	*N. tabacum*	Q40479
Pti4	*S. lycopersicum*	AAC50047

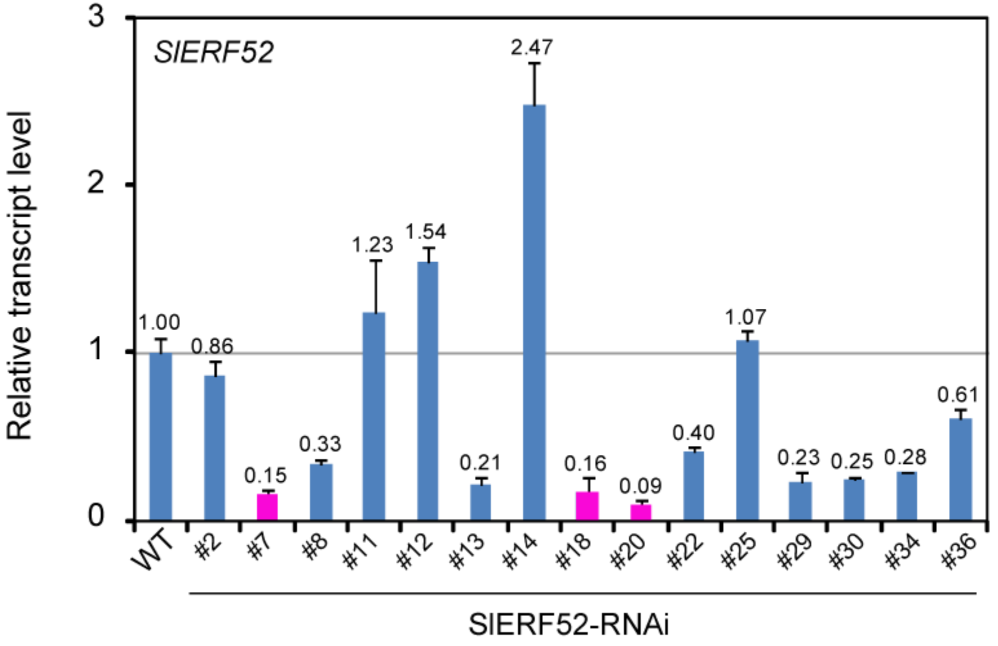

Figure S1. Expression analysis of SlERF52-RNAi transgenic plants.

Transcript levels of *SlERF52* in anthesis flower pedicles of 15 SlERF52-RNAi transgenic plants was measured by qRT-PCR. The transcript levels are shown as a fold-change value relative to that of wild-type (WT). The data were normalized to the expression of the *SAND* gene (SGN-U316474) as an internal control (Exposito-Rodriguez *et al.*, 2008). The RNA sample for each line was extracted from 3-10 pedicels. Error bars indicate standard error of technical replicates (n=2). Three plants in which *SlERF52* expression was greatly reduced were selected for further analyses (#7, #18 and #20, indicated in pink).

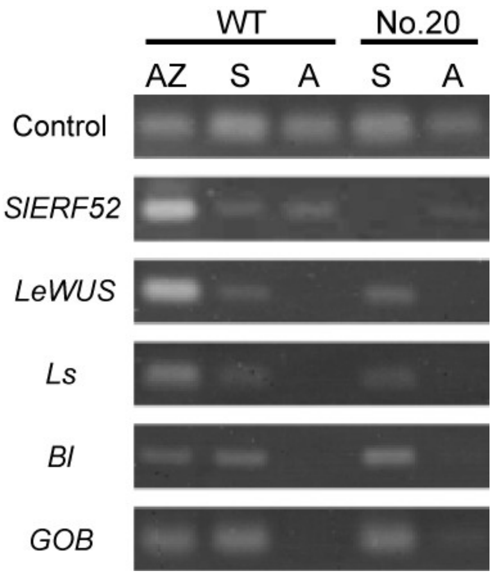

Figure S2. Expression of *SlERF52* and meristem-associated TF genes in shoot apex and leaf axilla.

Transcript levels of *SlERF52*, *LeWUS*, *Ls*, *Bl* and *GOB* were compared by RT-PCR. The expression levels of *LeWUS*, *Ls*, *Bl* and *GOB* in shoot apices of the wild type did not agree with the levels in leaf axillae, although *SlERF52* was expressed at similar levels in the two tissues. The *SlERF52*-RNAi transgenic plant (No.20) showed expression of *LeWUS*, *Ls*, *Bl* and *GOB* in the shoot apex at similar levels to wild type. These results suggest that the transcriptional regulation of these four genes in shoot apices was independent of *SlERF52*, unlike in AZs. AZ, abscission zone; S, shoot apex; A, leaf axila.
